# Curvature-based interface restoration algorithm using phase-field equations

**DOI:** 10.1371/journal.pone.0295527

**Published:** 2023-12-14

**Authors:** Seunggyu Lee, Yongho Choi

**Affiliations:** 1 Division of Applied Mathematical Sciences, Korea University, Sejong, Republic of Korea; 2 Biomedical Mathematics Group, Institute for Basic Science, Daejeon, Republic of Korea; 3 Department of Computer & Information Engineering, Daegu University, Gyeongsan, Republic of Korea; State University of New York at Binghamton: Binghamton University, UNITED STATES

## Abstract

In this study, we propose a restoration algorithm for distorted objects using a curvature-driven flow. First, we capture the convex-hull shaped contour of the distorted object using the mean curvature flow. With the supplemented mass on the captured feature, we evolve the constraint mean curvature flow to a steady state, preserving the non-distorted region. With respect to the mass, we select a restorative shape by considering the square of the curvature derivative. The Allen–Cahn and Cahn–Hilliard equations are applied to the generated restored image from the implicit curvature motions represented by the order parameter. We impose the Dirichlet boundary condition for the order parameter and the Neumann boundary for the chemical potential to fix the feature and to inherit the mass conservation, respectively. We provided examples of the restoration of half-circle and parentheses-shaped objects to reconstruct a circle shape.

## Introduction

Image data in electronic formats can be damaged in storage or transmission [1]. In medical tomography, imaging scans can be easily distorted by artificial effects, causing interference and interruption [2]. For example, in surgical imaging, a part of a medical image showing the skeleton could be accidentally damaged, which might cause difficulty in distinguishing the characteristic signs of some conditions or diseases [3]. Therefore, considerable effort has been devoted to the study of the reconstruction of distorted images.

**Fig 1:**
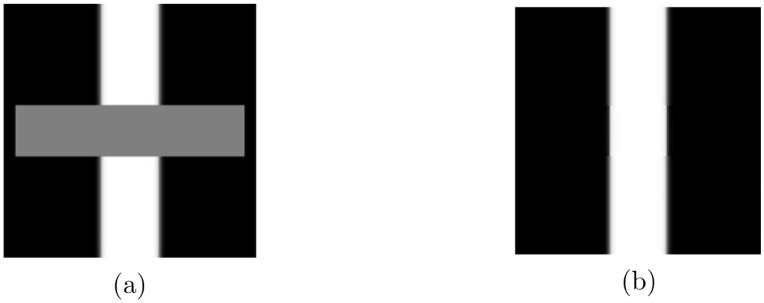
Image inpainting for (a) a damaged image and (b) image restoration. Reprinted from [9] with permission from Elsevier (Digital Signal Processing).

Image inpainting techniques reconstruct image data using information around damaged parts. Fig 1 shows a well-known 2D schematic of a conventional method to generate gray values in part of a vacant region. Fig 1(a) shows a given image containing a missing domain, and Fig 1(b) shows an image reconstructed using an image inpainting technique. Many models have been proposed to obtain a reasonable reconstruction [4–8] based on modifications of the Allen–Cahn and Cahn–Hilliard equations [9–11]. These successive binary image inpainting methods have been extended to a wide variety of applications [12–15] such as colored images and slice data in three-dimensional problems.

**Fig 2:**
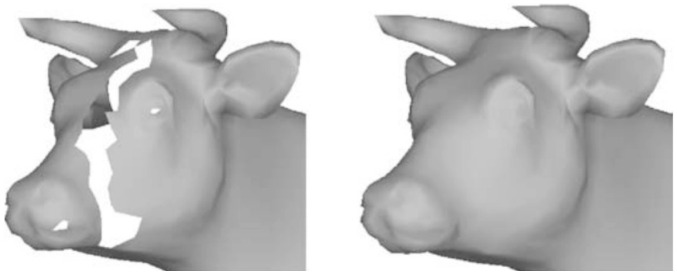
Hole-filling algorithm to reconstruct a damaged hole in a surface mesh. Reprinted from [19] with permission from Elsevier (Computer-Aided Design).

By contrast, an image domain (surface mesh) could be destroyed along with the image value, as shown in Fig 2. This has been investigated in terms of hole-filling or surface inpainting. In 3D cases, a volumetric reconstruction may be considered. However, in the present work, we discuss only surface restoration [16–23]. We refer interested readers to a survey of the relevant literature [24]. In [16], volumetric diffusion method was proposed as an algorithm to restore a complex surface. Moreover, several methods have been proposed that considered geometric properties such as curvature [17, 18, 21, 23]. In [19], a mesh generation and self-intersection from the projected boundary points on a plane were used to update surface mesh points inside a hole in a piecewise manner. In [20], an advancing front mesh algorithm was applied to fill in a hole, and the Poisson equation was used to heal over inside points. In [22], a sparsity-based hole-filling algorithm was proposed to find a low-dimensional intrinsic structure from a Laplacian eigenbasis dictionary.

**Fig 3:**
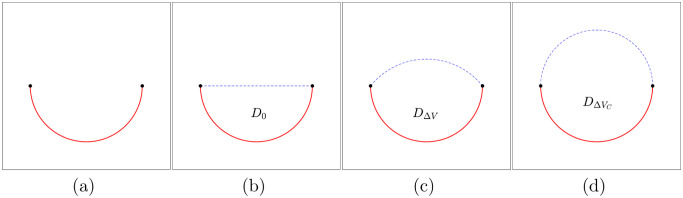
Interface restoration; (a) open curve, (b) closed region *D*_0_ obtained by filling the boundary with an area of *V*_0_, (c) curvature minimization shape *D*_Δ*V*_ with an area of *V*_0_ + Δ*V*, (d) selected result with a reasonable area of *V*_0_ + Δ*V*_*C*_.

In comparison, to distinguish surface restoration with image inpainting, the latter indicates the reconstructing of function values at grid points in the damaged region (in the case that we already know the locations of the grid points on a flatted 2D image), whereas the former reconstructs grid points for a damaged curved 2D image. To discuss this clearly, we consider the concept of interface restoration, which aims to recover a parametric curve from a destroyed curve. For example, given the open curve with two endpoints in Fig 3(a), we aim to find the closed curve that links the two points, where we assume that the original is closed, smooth, and non-twisted.

In this study, we propose a curvature-based interface restoration method. The desired curve is selected by the minimized curve of the curvature variation. We provide the proposed algorithm with a simple example in which a circle shape is partially lost. For example, refer to Fig 3. First, we denote the domain generated by connecting two points as *D*_0_, and its area by *V*_0_. Moreover, we define *D*_Δ*V*_ as the curvature minimization shape with the area of *V*_0_ + Δ*V*. Then, we select the desired curve by considering the curvature variation with respect to the increment of volume Δ*V*.

We employ the Allen–Cahn (AC) equation *ϕ*_*t*_ = −*μ* and the Cahn–Hilliard (CH) equation *ϕ*_*t*_ = Δ*μ*, where *μ* = *ϕ*^3^ − *ϕ* − *ϵ*^2^Δ*ϕ* is the chemical potential. These equations are considered to use the properties of curvature-driven flows [25–27], which were originally introduced to describe motion of interfaces in materials [28, 29]. Various studies are being conducted on the AC equation that conserves mass [30]. This can be used instead of the CH equation, but since the characteristics of the two equations are slightly different [31], so the CH equation is used in this research. In addition, we define a narrow band shape to represent the given damaged curve and the outer boundary to approximate the desired restored curve. We consider the Dirichlet boundary condition for both the AC and CH equations to fix the boundary near the undamaged curve, and the Neumann boundary condition for the CH equation to preserve the supplemented area. We described the detailed algorithm in the Numerical method section.

The remainder of this study is organized as follows. In the Numerical method section, we present the proposed numerical method with restoration algorithms defining capture and restorations step. In the Numerical results section, we describe the results of numerical simulations including half-circle and parentheses shapes. Finally, Conclusion section presents a discussion of our conclusions.

## Numerical method

**Fig 4:**
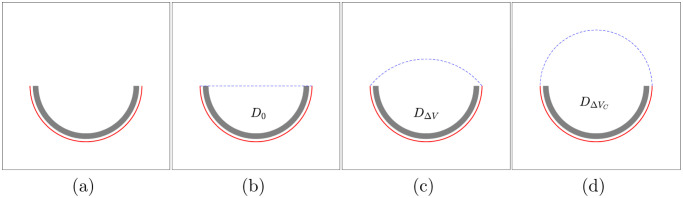
Interface restoration using the phase field equations; (a) gray obstacle to fix the given shape, (b) steady-state shape evolving AC equation, (c) steady-state shape evolving CH equation with an additional area Δ*V*, and (d) selected shape.

In this section, we describe each step in interface restoration in detail. The overall process is shown in Fig 4. First, we consider a narrow band to approximate the given shape in Fig 4(a). The steady-state solution of the AC equation is the shape of *D*_0_ in Fig 4(b), and we calculate its area *V*_0_, which is referred to as the capture step. Evolving the CH equation with the supplemented area *V*_0_ + Δ*V*, we obtain a steady-state solution *D*_Δ*V*_, referred to as the reconstruction step in Fig 4(c). Finally, we select a reasonable shape $D_{{\Delta V}_{C}}$ depending on the curvature derivation in Fig 4(d).

The proposed restoration algorithm consists of the following two sub-steps. The first step (capture) identifies the overall structure, including the missing part, and the second step (reconstruction) restores the shape through curvature minimization.

1. The capture step sets the order parameter ϕ(x,t) to capture a given damaged feature by using the AC equation with one Dirichlet boundary condition.
2. The reconstruction step reconstructs an implicit surface from ϕ(x,t) obtained in the previous step under the given volume *V*_0_ from the captured feature by using CH equations with one-Dirichlet and zero-Neumann boundary conditions.

The given damaged and captured features are respectively represented by the boundary control function G(x) in the capture and reconstruction steps. A boundary control function was investigated to solve PDEs numerically in complex domains [32–36]. In the present work, we use the boundary control function in a surface restoration algorithm with a proper boundary condition.

**Fig 5:**
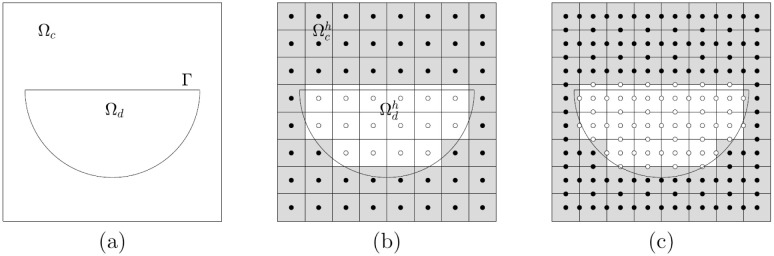
(a) Ω_*c*_ is a computational domain. (b) $\Omega_{c}^{h}$ is represented by closed circles, and (c) *G* = 1 at closed circles and *G* = 0 at open circles.

Let Ω = (0, *L*_*x*_) × (0, *L*_*y*_) be a domain, Ω_*c*_ ⊂ Ω be a computational domain, Ω_*d*_ ⊂ Ω be the domain of the given damaged feature, and Γ be the boundary between Ω_*c*_ and Ω_*d*_ (See Fig 5(a)). Here, the boundary control function is considered in a discrete sense to represent each domain on the set of the cell-centered grid points $G^{h}=\left\{x_{ij}=\left(\left(i-0.5\right)h,\left(j-0.5\right)h\right):i=1,\ldots ,N_{x},\;j=1,\ldots ,N_{y}\right\}$ with the space step size *h* and the number of grid points *N*_*x*_ = *L*_*x*_/*h* and *N*_*y*_ = *L*_*y*_/*h* in the *x*- and *y*-directions, respectively. Furthermore, let $\Omega_{c}^{h}=\Omega_{c}\cap G^{h}$ and $\Omega_{d}^{h}=\Omega_{d}\cap G^{h}$ (See Fig 5(b)). Then, the discrete boundary condition function *G*_*ij*_ at $G^{h}$ is defined as follows.

$$\begin{array}{c} G_{ij}=\{\begin{array}{cc} 1, & \text{if}\;x_{ij}\in \Omega_{c}^{h},\\ 0, & \text{otherwise}. \end{array} \end{array}$$ (1)

Moreover, we extend the definition of *G*_*ij*_ to the cell-edged grid points as follows (See Fig 5(c)).

$$\begin{array}{c} G_{i+\frac{1}{2},j}=G_{ij}G_{i+1,j},\;G_{i,j+\frac{1}{2}}=G_{ij}G_{i,j+1}. \end{array}$$ (2)

### Capture step

The governing equation of the capture step is the AC equation with one Dirichlet boundary condition for ϕ(x,t), as follows.

$$\begin{array}{c} \frac{\partial \phi (x,t)}{\partial t}=-\frac{{\left(\phi \left(x,t\right)\right)}^{3}-\phi \left(x,t\right)}{\epsilon^{2}}+\Delta \phi \left(x,t\right)\;\text{for}\;x\in \Omega_{c}, \end{array}$$ (3)

$$\begin{array}{c} \phi (x,t)=1\;\text{for}\;x\in \Gamma , \end{array}$$ (4)

where *ϵ* is the coefficient related with an interface thickness. The initial condition ϕ(x,0) is satisfied in that its values are defined as 1 for $x\in \Omega_{d}$ and its zero-contour covers Ω_*d*_.

At this point, (3) and (4) can be discretized on $G^{h}$ as follows.

$$\begin{array}{c} \frac{\phi_{ij}^{n+1}-\phi_{ij}^{n}}{\Delta t}=G_{ij}(-\frac{{\left(\phi_{ij}^{n}\right)}^{3}-\phi_{ij}^{n}}{\epsilon^{2}}+\frac{\phi_{i+1,j}^{n}+\phi_{i,j+1}^{n}-4\phi_{ij}^{n}+\phi_{i-1,j}^{n}+\phi_{i,j-1}^{n}}{h^{2}}), \end{array}$$ (5)

$$\begin{array}{cc} \phi_{ij}^{n} & =1\;\text{for}\;x_{ij}\in \Omega_{d}^{h}, \end{array}$$ (6)

where $\phi_{ij}^{n}$ is the approximations of $\phi (x_{ij},n\Delta t)$, and Δ*t* is the time step size.

The capture step is complete when $\phi_{ij}^{n}$ reaches a steady state.

### Reconstruction step

The reconstruction step is governed by the types of the CH equations using the boundary control function

$$\begin{array}{c} \frac{\partial \phi (x,t)}{\partial t}=\Delta \mu \left(x,t\right)\;\text{for}\;x\in \Omega_{c}, \end{array}$$ (7)

$$\begin{array}{c} \mu (x,t)={\left(\phi \left(x,t\right)\right)}^{3}-\phi \left(x,t\right)-\epsilon^{2}\Delta \phi \left(x,t\right), \end{array}$$ (8)

with one-Dirichlet and zero-Neumann boundary conditions for *ϕ* and *μ*, respectively.

$$\begin{array}{c} \phi \left(x,t\right)=1\;\text{and}\;\frac{\partial \mu (x,t)}{\partial n}\left(x\right)=0\;\text{for}\;x\in \Gamma . \end{array}$$ (9)

Here, the initial condition is set by the steady-state solution of the capture step.

The discretization of the system (7) and (9) on $G^{h}$ can be expressed as follows.

$$\begin{array}{c} \frac{\phi_{ij}^{n+1}-\phi_{ij}^{n}}{\Delta t}=\nabla_{d}\cdot {\left(G\nabla_{d}\mu^{n}\right)}_{ij}, \end{array}$$ (10)

$$\begin{array}{c} \mu_{ij}^{n}=G_{ij}[{\left(\phi_{ij}^{n}\right)}^{3}-\phi_{ij}^{n}-\epsilon^{2}(\frac{\phi_{i+1,j}^{n}+\phi_{i,j+1}^{n}-4\phi_{ij}^{n}+\phi_{i-1,j}^{n}+\phi_{i,j-1}^{n}}{h^{2}})], \end{array}$$ (11)

where

∇d·(G∇dμn)ij=Gi+12,j(μi+1,jn-μijn)-Gi-12,j(μijn-μi-1,jn)h2=+Gi,j+12(μi,j+1n-μijn)-Gi,j-12(μijn-μi,j-1n)h2. (12)

Because the summation by parts is satisfied for $\mu_{ij}^{n}$ [36], discrete mass conservation holds as follows.

$$\begin{array}{c} \begin{array}{cc} {\left(\phi^{n+1},1\right)}_{h} & ={\left(\phi^{n},1\right)}_{h}+\Delta t(\nabla_{d}\cdot \left(G\nabla_{d}\mu^{n}\right),1)\\ & ={\left(\phi^{n},1\right)}_{h}-\Delta t(G\nabla_{d}\mu^{n},\nabla_{d}1)={\left(\phi^{n},1\right)}_{h}. \end{array} \end{array}$$ (13)

## Numerical results

We set the tolerance for the numerical simulation as *tol* = 0.5 ⋅ 10^−6^.

### Grid-independent test

**Fig 6:**
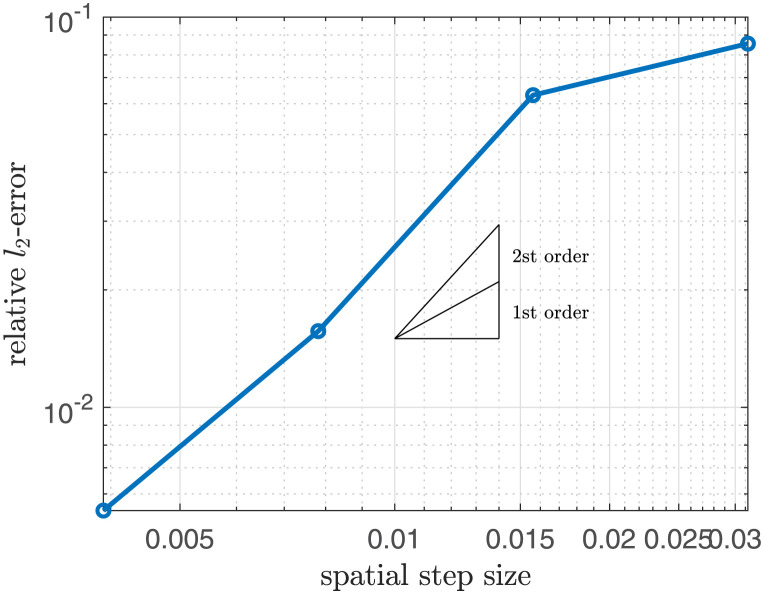
Relative *l*_2_-error with respect to spatial step size.

To confirm our method, we first perform the numerical simulation that gives independency of the number of grid points before full restoration steps. Here, we set a zero-Neumann boundary condition and the initial condition

$$\begin{array}{c} \psi (x,y,0)=\text{tanh}(\frac{d(x,y)}{\sqrt{2}\epsilon }), \end{array}$$ (14)

where $d\left(x,y\right)=0.35-\sqrt{{\left(x-0.5\right)}^{2}+{\left(y-0.5\right)}^{2}}$ on the two-dimensional domain Ω = [0, 1] × [0, 1]. The obstacle was defined as the middle of hemicircles with radii of 0.2 and 0.24. The center of the circles is the center of the computational domain. Fig 6 shows the relative *l*_2_-error ‖*ϕ*(*x*, *y*, *T*) − *ref*(*x*, *y*)‖_2_/‖*ref*(*x*, *y*)‖_2_ where Δ*t* = 10^−8^ and *T* = 0.2 with various numbers of grid point *N*_*x*_ = 2^*k*^ for *k* = 5, …. 10. The slope of graph represents the second-order accuracy consistenting with the theory in [32]. Moreover, it indicates a grid-independency that the scaling of error is smaller than 10^−1^ in spite of the small number of the grid point.

### Half-circle shape

**Fig 7:**
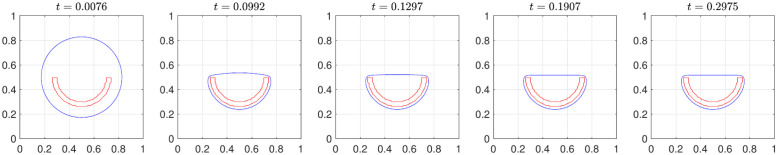
Evolution of the zero-contour for AC equation.

We begins by evolving the solution of the AC equation with a zero-Neumann boundary condition and the initial condition same as above. Fig 7 shows the evolution of the solution of the AC equation in blue and the obstacle shape in red. Because the AC equation can be explained as the mean curvature flow, the initial circle rapidly shrinks before the iso-contour contacts the given obstacle. Then, they evolve into half-circle shape, preserving the convex hull property.

Next, using the steady-state solution $\tilde{\psi }$ from the AC equation, we evolve the CH equation with the same obstacle to the steady state. For the numerical simulation of a desired volume *V*_*β*_ with a parameter of *β*, we set an initial condition

$$\begin{array}{c} \phi (x,y,0)=1+\tilde{\psi }\left(x,y\right)+\text{tanh}(\frac{d(x,y)}{\sqrt{2}\epsilon }), \end{array}$$ (15)

where $d\left(x,y\right)=(r-\sqrt{{\left(x-0.5\right)}^{2}+{\left(y-0.5-r-4\Delta y\right)}^{2}}$, $r=\sqrt{\beta (V_{\beta }+V_{0})/\left(2\pi \right)}$, and $V_{0}=\text{mean}\left(\tilde{\psi }\right)$. With the initial condition (15), we consider the steady-state solution of the conservative mean curvature flow with the specific volume *V*_*β*_.

**Fig 8:**
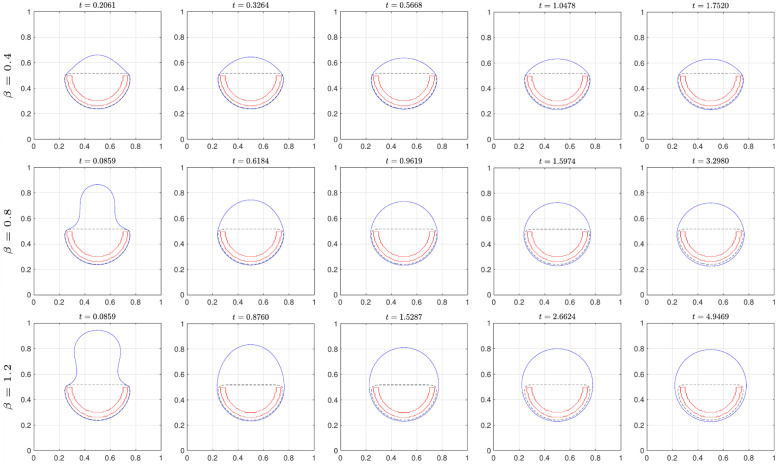
Evolution of the zero-contour for CH equation with various *β*.

Fig 8 shows the evolution of the solution for the CH equation with several parameters of *β*. The last column indicates the numerical steady-state solution such that the consecutive error of the solution is less than *tol*. The red-solid and black-dashed curves represent the obstacle and the final solution $\tilde{\psi }$ for the AC equation, respectively. Owing to the nature of the mean curvature flow, the iso-contour of the solution for CH equation near the obstacle tends to remain while the other region is searched for the optimized circle-like shape. With increasing values of *β*, the final solutions converge on the intended circle.

**Fig 9:**
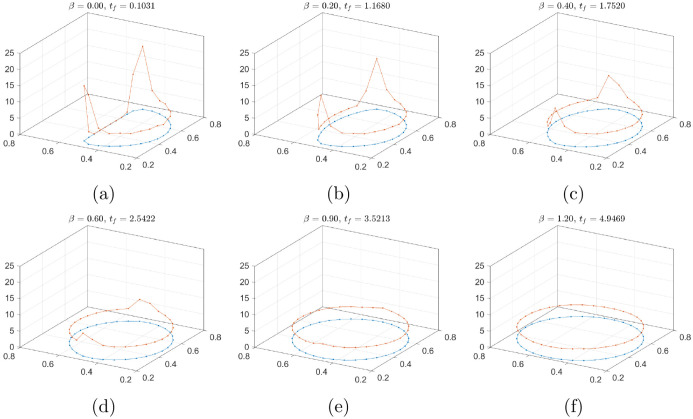
Curvature values on the zero-contour at the final time. The corresponding values of $D_{\kappa }$: (a) 10900, (b) 5190, (c) 1750, (d) 330, (e) 8.85, and (f) 1.14.

To investigate the curvature distribution with respect to the volume, we compute the curvature on the zero-contour of the steady-state solution with various *β*. To numerically compute the curvature, we generate the contour points using the DistMesh algorithm [37]. Fig 9 shows the curvature plots for the various values of *β*. The blue line shows the zero-contour of the final solution at *t* = *t*_*f*_, and the red line represents the curvature value on the zero-contour. With increasing *β*, the distribution of curvature is smeared and the high magnitude near the endpoints decrease.

To characterize the steady-state shape using the curvature distribution, we consider the integration of the square of curvature derivative as

$$\begin{array}{c} D_{\kappa }=\int_{\Gamma }{∥\frac{d\kappa }{ds}∥}^{2}ds. \end{array}$$ (16)

**Fig 10:**
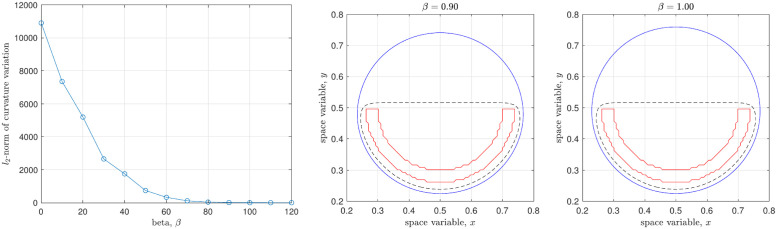
Curvature variation $D_{\kappa }$ with respect to *β* and the selectable final solutions.

The corresponding values of $D_{\kappa }$ of Fig 9 are 10900, 5190, 1750, 330, 8.85, and 1.14, respectively. Fig 10 shows the curvature variation $D_{\kappa }$ with the various *β*, the value of which monotonically decreases. It should be noted that choice of a too large area *V*_0_(1 + *β*) could make an interface diverge from the target shape in spice of the obstacle and the Dirichlet boundary condition. Therefore, a standard is required to make a reasonable choice, and we thus adopt the steady-state solution for *β* = 0.9 and *β* = 1.

### Parentheses shape

We begin by evolving the solution of the AC equation with a zero-Neumann boundary condition and the initial conditions, as given below.

$$\begin{array}{c} \psi (x,y,0)=\text{tanh}(\frac{d(x,y)}{\sqrt{2}\epsilon }), \end{array}$$ (17)

where $d\left(x,y\right)=0.35-\sqrt{{\left(x-0.5\right)}^{2}+{\left(y-0.5\right)}^{2}}$ on the two-dimensional domain Ω = [0, 1] × [0, 1]. The obstacle is defined as the middle of parentheses shapes describing parts of a circle with radii of 0.2 and 0.24. The center of the circles is the center of the computational domain.

**Fig 11:**
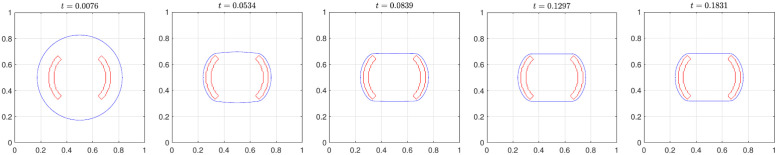
Evolution of the zero-contour for AC equation.

Fig 11 shows the evolution of the solution for the AC equation in blue and the obstacle shape in red. Similarly, for the results of the previous section, the initial circle rapidly shrinks before the iso-contour contacts the given obstacle and then evolves into the tablet shape, preserving the convex hull property.

Next, using the steady-state solution $\tilde{\psi }$ from the AC equation, we evolve the CH equation with the same obstacle to the steady state. For the numerical simulation of a desired volume *V*_*β*_ with a parameter of *β*, we set an initial condition

$$\begin{array}{c} \phi (x,y,0)=1+\tilde{\psi }\left(x,y\right)+\text{tanh}(\frac{d_{1}\left(x,y\right)}{\sqrt{2}\epsilon })+\text{tanh}(\frac{d_{2}\left(x,y\right)}{\sqrt{2}\epsilon }), \end{array}$$ (18)

where

$$\begin{array}{cc} d_{1}\left(x,y\right) & =r-\sqrt{{\left(x-0.5\right)}^{2}+{\left(y-0.7-0.5r-4\Delta y\right)}^{2}}, \end{array}$$ (19)

$$\begin{array}{cc} d_{2}\left(x,y\right) & =r-\sqrt{{\left(x-0.5\right)}^{2}+{\left(y-0.3+0.5r+4\Delta y\right)}^{2}}, \end{array}$$ (20)

$r=\sqrt{\beta (V_{\beta }+V_{0})/\left(4\pi \right)}$, and $V_{0}=\text{mean}\left(\tilde{\psi }\right)$. With the initial condition (18), we consider the steady-state solution of the constraint mean-curvature flow with the specific volume *V*_*β*_.

**Fig 12:**
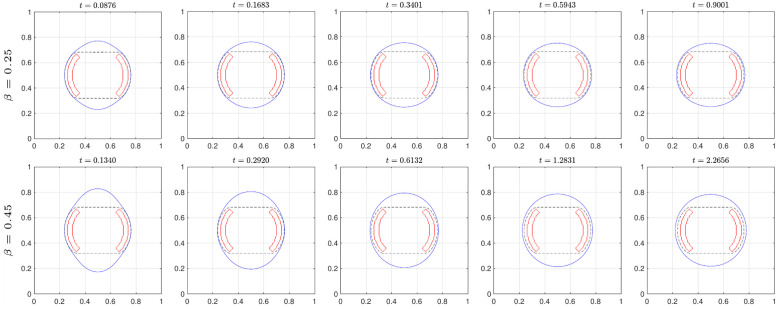
Evolution of the zero-contour for CH equation with various *β*.

Fig 12 shows the evolution of the solution for the CH equation with several parameters for *β*. The last column indicates the numerical steady-state solution such that the consecutive error of the solution is less than *tol*. The red-solid and black-dashed curves represent the obstacle and the final solution $\tilde{\psi }$ for the AC equation, respectively. Owing to the nature of the mean curvature flow, the iso-contour of the solution for the CH equation near the obstacle tends to remain, and the other regions are searched for the optimized circle-like shape. By increasing the value of *β*, the final solutions converge to the circle.

**Fig 13:**
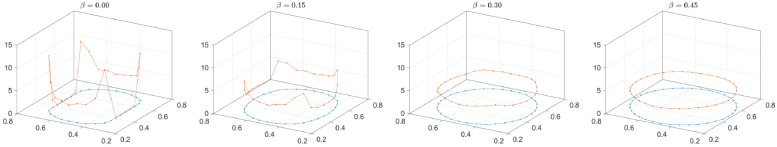
Curvature values on the zero-contour at the final time.

Fig 13 shows the curvature plots for the selected parameters of *β*. The blue line is the zero-contour of the final solution at *t* = *t*_*f*_ and the red line presents the curvature value. The corresponding values of $D_{\kappa }$ are 6350, 752, 14.5, and 1.30, respectively.

**Fig 14:**
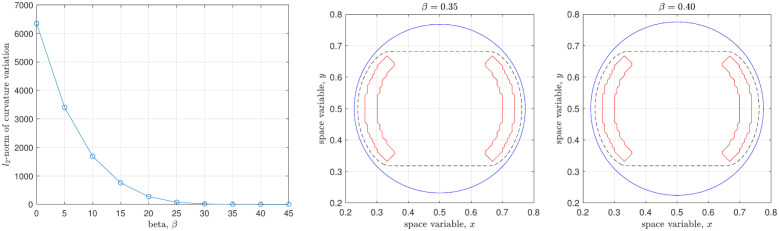
Curvature derivation $D_{\kappa }$ with respect to *β* and the selected final solutions.

As shown in Fig 14, the curvature variation $D_{\kappa }$ monotonically decreases with the various *β*, as shown in as Fig 10. In this case, the steady-state solutions for *β* = 0.35 and *β* = 0.4 appear to be the reasonable choices.

### Skull shape in CT image

**Fig 15:**
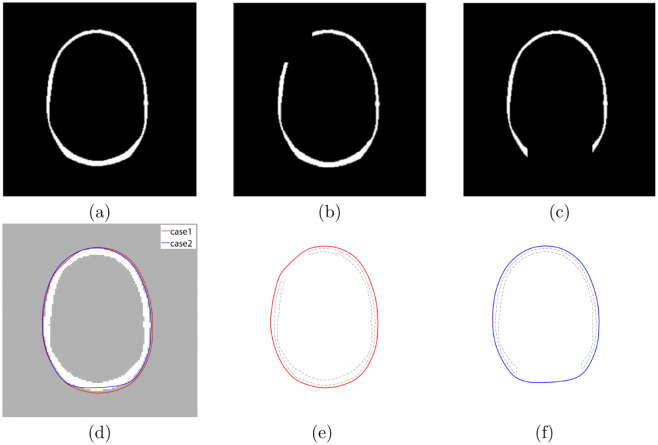
Comparison of the restoration results: (a) original skull image, (b) fromt left cut out [case1], (c) back of the head cut out [case2], (d) comparison result [red and blue line are our results], (e) red line is our result for case1, and (f) blue line is our result for case2. Reprinted from [38] with permission from Springer Nature (Journal of Medical Systems).

The previous research [38], while conducting research on the development of skull protheses through an active contour model, restored the part of the skull if it takes damaged (missing) part from the corresponding CT image (skull). To confirm our proposed algorithm, we also choose the same CT image (Fig 15(a)) and perform the restoration with two different damaged scenarios: front left cut out [case 1] in Fig 15(b) and back of the head cut out [case 2] in Fig 15(c). The numerical results are shown in Fig 15(d)–15(f). Note that both results have good agreements with the original skull shape. We upload the basic data and visualization code for this simulation to the corresponding author’s web page (https://sites.google.com/view/yh-choi/code) and DRYAD site (DOI: 10.5061/dryad.fbg79cp2g).

## Conclusion

In this study, we proposed a restoration algorithm for a distorted object using curvature-driven flows. We captured the outer contour using the Allen–Cahn equation with the one-Dirichlet boundary condition for the distorted object. Subsequently, we evolved the Cahn–Hilliard equation to a steady state with the supplemented mass on the captured feature. Imposing the one-Dirichlet boundary condition for the order parameter and zero-Neumann boundary condition for the chemical potential, we fixed the feature and inherited the mass conservation. Finally, we chose the restored shape by considering the square of the curvature derivative. We provided two restoration examples for half-circle and parentheses objects showing how the proposed approach reconstructed a circle shape. The numerical results showed that the curvature distribution became more diffused with increasing supplemented mass, and it converged to the circle shape. The approximated retoration gave expected results; however, it may be broken when we set the too large mass since the one-Dirichlet boundary condition cannot exactly capture the fixed endpoint. We leave the problem of fixing the endpoints as a subject for future work.

## Supporting information

S1 Dataset (ZIP)
