# Supplementary figures and images for: Curvature-based interface restoration algorithm using phase-field equations
Source: PLoS One. 2023 Dec 14;18(12):e0295527. doi: 10.1371/journal.pone.0295527 (PMC10721061; doi:10.1371/journal.pone.0295527)

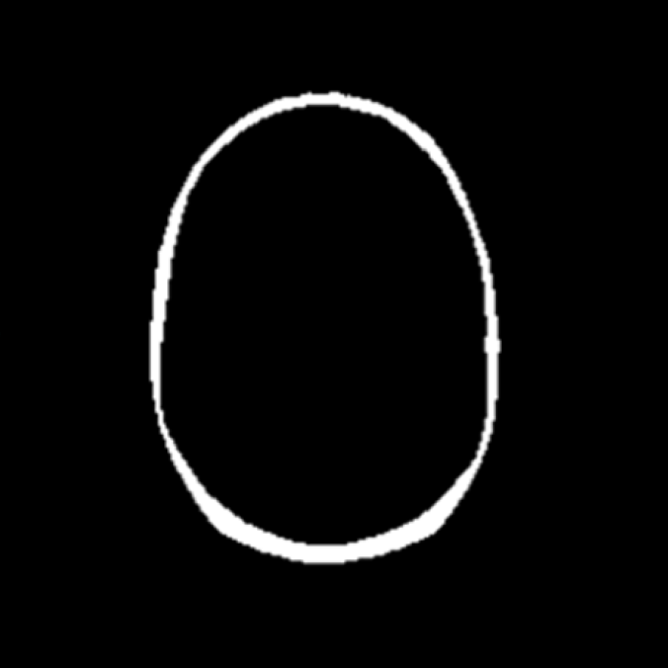

Supplement: S1 Dataset — (ZIP) [file pone.0295527.s001.zip › HEAD_image0.png]
